# Upregulation of SNAP25 by HDAC inhibition ameliorates Niemann‐Pick Type C disease phenotypes via autophagy induction

**DOI:** 10.1002/ctm2.776

**Published:** 2022-04-05

**Authors:** Yooju Jung 1,‡, Seung‐Eun Lee 2,‡, Insung Kang, Sung Min Cho, Kyung‐Sun Kang, Ho Jeong Kwon

**Affiliations:** ^1^ Chemical Genomics Leader Research Laboratory Department of Biotechnology College of Life Science and Biotechnology Yonsei University Seoul Republic of Korea; ^2^ Adult Stem Cell Research Center and Research Institute for Veterinary Science College of Veterinary Medicine Seoul National University Seoul Republic of Korea

Dear Editor

Niemann‐Pick Type C disease (NPC) is a rare fatal neurodegenerative disorder caused by mutations in the *NPC1* or *NPC2* gene,^1^ leading to abnormal accumulation of non‐esterified cholesterol in lysosomes. Defective autophagy caused by the failure of autolysosome formation composed of SNARE machinery^2^ has been reported in NPC disease and synaptosomal‐associated protein 25 (SNAP25) has been highlighted as one of the important components of SNARE machinery.^3^, ^4^ Currently, there are no Food and Drug Administration (FDA)‐approved treatments and a recent study shows that histone deacetylase (HDAC) inhibitors may be promising therapeutics for NPC disease.^5^

To investigate the effects of HDAC inhibition in NPC‐iNSCs, suberoylanilide hydroxamic acid (SAHA), *N*‐hydroxy‐7‐(2‐naphthylthio) heptanomide (HNHA), FK228, and valproic acid (VPA) were treated at a non‐toxic concentration (Figure S1A–E) and non‐esterified cholesterol was stained with filipin III (Figure 1A,B). HDAC inhibitor‐treated NPC‐iNSCs had lower levels of non‐esterified cholesterol and among them, HNHA showed the most effective cholesterol reduction. Likewise, the free cholesterol level was notably reduced by the HDAC inhibitor‐treatment including SAHA which has previously exhibited an ameliorated activity in NPC disease^6^ together with a new synthetic HDAC inhibitor, HNHA^7^ (Figure 1C).

**FIGURE 1:**
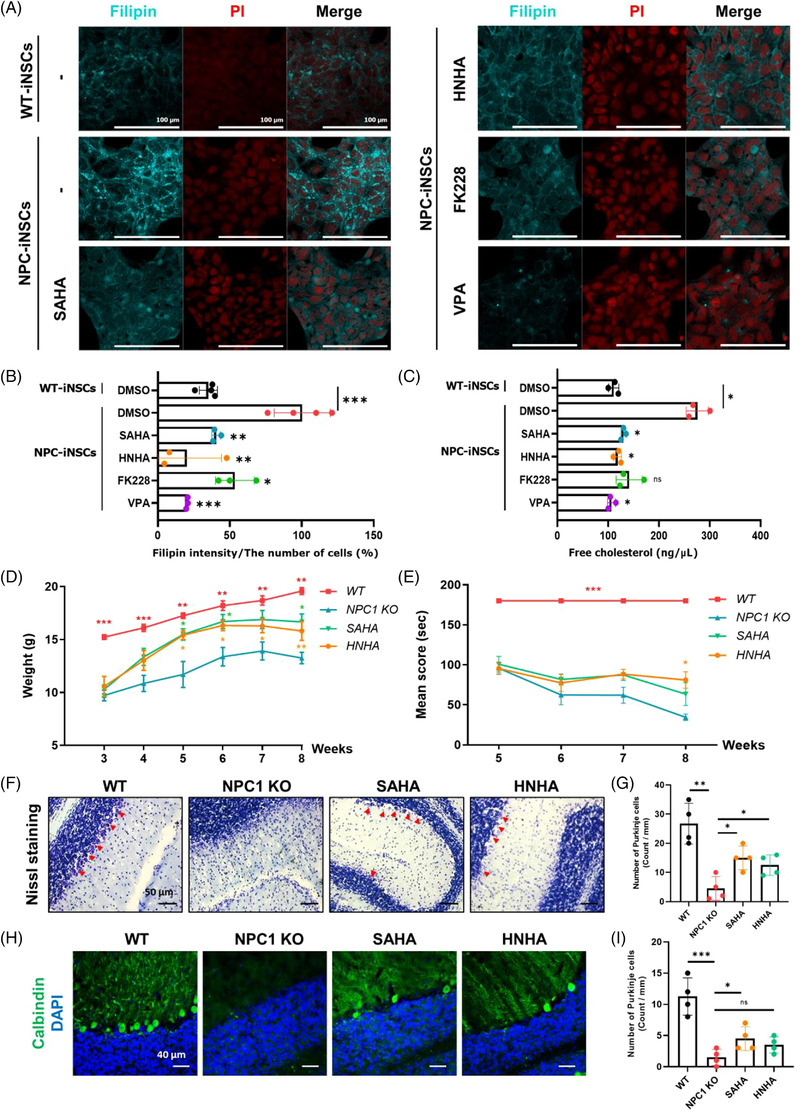
Treatment of two HDAC inhibitors, suberoylanilide hydroxamic acid (SAHA) and *N*‐hydroxy‐7‐(2‐naphthylthio) heptanomide (HNHA), reduced non‐esterified cholesterol in Niemann‐Pick Type C disease (NPC)‐iNSCs and NPC1 KO mice. (A) NPC‐iNSCs were treated with SAHA (1 μM), HNHA (1 μM), FK228 (10 nM), tubacin (10 nM), or VPA (1 mM) for 48 h and then stained with filipin III. Scale bar, 100 μm. (B) The density of filipin‐positive area was quantified, and the value was standardised using ImageJ. Graph shows the means ± SD (*n* ≥ 3). (C) NPC‐iNSCs were treated with SAHA (1 μM), HNHA (1 μM), FK228 (10 nM), VPA (1 mM) for 48 h and then processed for cholesterol assay. The graph shows the means ± SD (*n* = 3). (D) Body weight in WT, NPC1 KO, SAHA‐ and HNHA‐administrated group during the 5 weeks of treatment. (E) Rotarod testing was measured using the accelerating rotarod test (30 rpm/min) in WT, NPC1 KO, SAHA‐ and HNHA‐administrated group during the 5 weeks of treatment. (F) Images of Nissl positive cells (red arrows) in cerebellar of WT, NPC1 KO mice, SAHA‐ and HNHA‐treated groups at 8 weeks of age. Scale bar, 50 μm. (G) Quantification of Nissl‐positive cells in the cerebellum of WT, NPC1 KO mice, SAHA‐ and HNHA‐treated groups at 8 weeks of age. (H) Representative images of calbindin positive cells in cerebellar of WT, NPC1 KO mice, SAHA‐ and HNHA‐treated groups at 8 weeks of age. Scale bar, 40 μm. (I) Quantification of purkinje cells across all cerebellar lobules from four mice (four sections per mouse) is shown in the bar graph (represented as a fraction of purkinje cells relative to control healthy mice). Statistical significance was assessed by Student's *t*‐test. ***P < 0.001; **P < 0.01; *P < 0.05

We then compared the effects of the long‐term treatment of compounds in NPC1 KO mice. SAHA and HNHA (HDACi)‐treated mice showed improvement in body weight relative to vehicle‐treated NPC1 KO mice (Figure 1D). Furthermore, motor function monitored using rotarod tests exhibited improvement in HDACi‐treated NPC1 KO mice. Each HDACi‐treated NPC1 KO mice (*p* = 0.014) sustained the rotarod test for 100, 81, 87, 63 s and 95, 77, 88, 81 s at 5‐, 6‐, 7‐ and 8‐weeks post‐injection, whereas the vehicle‐treated NPC1 KO mice lasted for a shorter amount of time (95, 62, 62 and 34, respectively, Figure 1E). A pathological phenotype of NPC is the progressive death of cerebellar Purkinje cells,^8^ so we used Nissl‐ and calbindin‐positive staining to measure neurodegeneration of Purkinje cells. The total number of Nissl‐ or calbindin‐positive cells in the HDACi‐treated NPC1 KO mice increased 2.3‐fold (SAHA) and 1.8‐fold (HNHA) or 3‐fold (SAHA) and 2.3‐fold (HNHA) in the cerebella, respectively (Figure 1F–I).

Next, the mode of action of HDACi in NPC‐iNSCs was analysed through total gene expression analysis using RNA‐seq (Figure 2A–C). Heat maps showed differential expression of genes in wild type (WT), NPC and HDACi‐treated NPC‐iNSCs (Figure 2A). The 13 genes expressing the same tendency were selected in both HDACi‐treated groups compared to the dimethyl sulfoxide (DMSO)‐treated group (Figure 2B). Furthermore, we analysed gene profiles by using the evolutionary genealogy of genes: Non‐supervised Orthologous Groups (eggNOG) browser (Table 1). The top eight categories showing significant differences were selected and an in‐depth analysis of the factors for category U, intracellular trafficking, secretion, and vesicular transport, was conducted (Figure 2C). In the list of genes showing the greatest difference, SNAP25 increased the most (Table 2).

**FIGURE 2:**
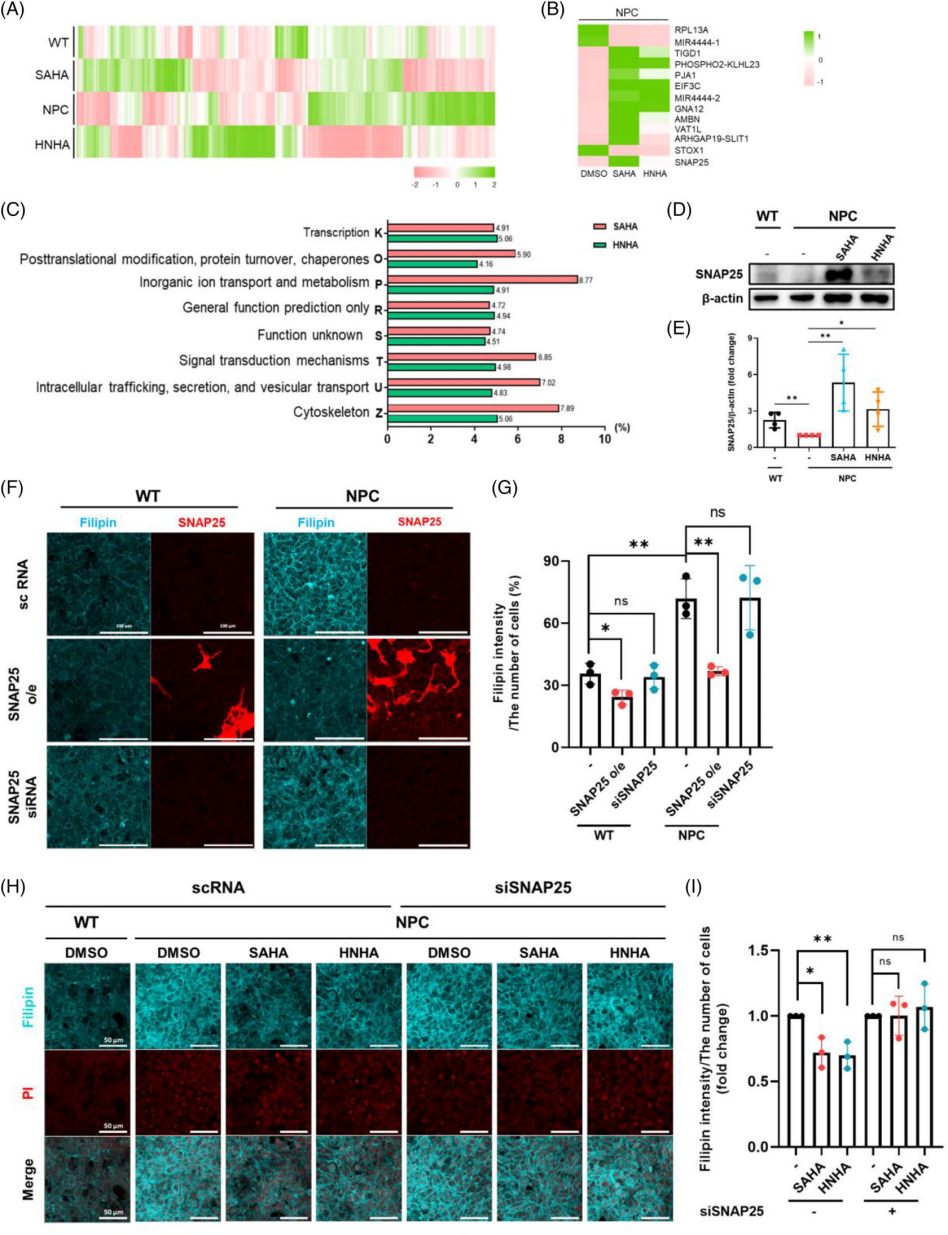
SNAP25 is upregulated in Niemann–Pick Type C disease (NPC)‐iNSCs after treatment with suberoylanilide hydroxamic acid (SAHA) and *N*‐hydroxy‐7‐(2‐naphthylthio) heptanomide (HNHA). (A) Heat map showing the clustering of total genes based on differential gene expression (*z*‐values) of WT, NPC+DMSO, NPC+SAHA and NPC+HNHA. (B) Heat map showing the clustering of 13 genes expressing the same tendency among the top 100 genes of both SAHA‐ and HNHA‐treated NPC‐iNSCs compared to DMSO‐treated NPC‐iNSCs. (C) Results from the eggNOG browser: K: Transcription; O: Post‐translational modification, protein turnover, chaperones; P: Inorganic ion transport and metabolism; R: General function prediction only; S: Function unknown; T: Signal transduction mechanisms; U: Intracellular trafficking, secretion, and vesicular transport; Z: Cytoskeleton. (D) The protein level of SNAP25 in NPC‐iNSCs treated with SAHA and HNHA for 48 h. (E) Immunoblot band intensity normalized to β‐actin expression. The graphs show the means ± SD (*n* = 4). (F) WT‐iNSCs and NPC‐iNSCs were transfected with SNAP25‐GFP overexpression transcripts or siRNA targeting SNAP25 for 48 h, then stained with filipin III. (G) The density of filipin‐positive areas was quantified, and the value was standardized using ImageJ. The graphs show the means ± SD (*n* = 3). (H) NPC‐iNSCs were transfected with siRNA targeting SNAP25 for 24 h, then SAHA (1 μM) and HNHA (1 μM) were treated for 48 h. Cells were stained with filipin III. (I) The density of the filipin‐positive area was quantified, and the value was standardised using ImageJ. The graphs show the means ± SD (*n* = 3). Statistical significance was assessed by Student's *t*‐test. ***P < 0.001; **P < 0.01; *P < 0.05

**TABLE 1:** Number of gene list of 26 functional categories of eggNOG browser

	**Functional categories**	**NPC‐SAHA**	**NPC‐HNHA**
		**(Number of genes/total number of genes)**	
J	Translation, ribosomal structure and biogenesis	4/462	2/462
A	RNA processing and modification	10/246	15/246
K	Transcription	64/1304	66/1304
L	Replication, recombination and repair	5/318	7/318
B	Chromatin structure and dynamics	11/271	10/271
D	Cell cycle control cell division, chromosome partitioning	9/212	5/212
Y	Nuclear structure	0/1	0/1
V	Defense mechanisms	3/45	3/45
T	Signal transduction mechanisms	66/964	48/964
M	Cell wall/membrane/envelope biogenesis	1/58	2/58
N	Cell motility	1/10	1/10
Z	Cytoskeleton	39/494	25/494
W	Extracellular stuructures	0/0	0/0
U	Intracellular trafficking, secretion, and vesicular transport	189/2691	130/2691
O	Posttranslational modification, protein turnover, chaperones	122/2068	86/2068
C	Energy production and conversion	11/240	8/240
G	Carbohydrate transport and metabolism	9/295	13/295
E	Amino acid transport and metabolism	19/273	11/273
F	Nucleotide transport and metabolism	9/124	3/124
H	Coenzyme transport and metabolism	3/76	0/76
I	Lipid transport and metabolism	14/320	6/320
P	Inorganic ion transport and metabolism	25/285	14/285
Q	Secondary metabolites biosynthesis, transport and catabolism	3/93	8/93
R	General function prediction only	187/3964	196/3964
S	Function unknown	184/3881	175/3881
NA	Not assigned	416/41412	849/41412

Abbreviations: HNHA, *N*‐hydroxy‐7‐(2‐naphthylthio) heptanomide; NPC, Niemann–Pick Type C disease; SAHA, suberoylanilide hydroxamic acid.

**TABLE 2:** Top genes of U category

**NPC‐SAHA**		**NPC‐HNHA**	
**Gene name**	**Fold change**	**Gene name**	**Fold change**
**SNAP25**	**37.714**	TMOD4	27.250
CD33	23.667	CX3CR1	15.000
GPR1	15.833	SPAG6	14.500
P2RY6	15.500	CD33	11.667
GRIN2A	15.000	GRID2	10.000
HLA‐DRA	14.646	P2RY6	9.500
LAMP5	14.500	CHRNG	9.250
EMR1	13.500	MTTP	8.400
KCNE1	12.667	RGPD1	8.000
RPRM	12.625	**SNAP25**	**7.429**
NCR3	11.900	KCNJ13	7.000
TRPC4	11.800	PROM1	7.000
GABRE	11.000	KCNE1	6.333
CX3CR1	11.000	CD74	5.800
SCN3A	10.500	UPK1A	5.800
SEC16B	10.000	PMCH	5.636
GPR12	10.000	NOX1	5.500

Abbreviations: HNHA, *N*‐hydroxy‐7‐(2‐naphthylthio) heptanomide; NPC, Niemann–Pick Type C disease; SAHA, suberoylanilide hydroxamic acid.

We confirmed that the protein levels of SNAP25 were increased by HDACi treatment (Figure 2D,E). In addition, overexpression of SNAP25 in NPC‐iNSCs decreased free cholesterol, demonstrating that upregulation of SNAP25 in NPC‐iNSCs can result in a reduction of lipids (Figure 2F,G). Each compound was then treated on both control and SNAP25 knocked down cells to compare the effect of compounds on SNAP25. Notably, the effect of compounds was significantly decreased when SNAP25 was silenced compared to when it was not (Figure 2H,I), implying that SNAP25 plays as a key target gene of HDACi treatment.

Referring to previous studies,^3^, ^4^ we hypothesized that the potential mechanism by which SNAP25 regulates cholesterol levels in NPC‐iNSCs might be through autophagy. We found that SNAP25 was deficient in NPC‐iNSCs (Figure S3A,B) and when SNAP25 was overexpressed in NPC‐iNSCs, p62, LC3‐I/II, and LAMP1a were markedly reduced (Figure 3A,B). Next, we examined whether SNAP25 directly interacts with the components of the SNARE complex by co‐immunoprecipitation analysis for endogenous SNAP25, and it was revealed that HDACi‐treatment increased the interaction between SNAP25, STX17, and VAMP8 in the SNARE complex (Figure 3C). A strong interaction between Vamp8‐SNAP25‐STX17 in HDACi‐treated NPC‐iNSCs was also verified by proximity ligation assay (PLA, Figure 3D). Although SNAP25 was increased by HDACi‐treatment compared to DMSO, Rapamycin (Rapa) and Bafilomycin (Baf) treatment did not decrease autophagy markers (Figure 3E,F). After Baf treatment, p62 and LC3‐Ⅱ were more accumulated and both markers were decreased by HDACi treatment which were administrated after Baf treatment while increasing SNAP25 (Figure S3E). Next, we used a tandem fluorescent‐tagged mRFP–GFP–LC3 reporter to assess autophagic flux.^9^ Autophagosomes (mRFP^+^–GFP^+^–LC3) were abnormally accumulated in NPC‐iNSCs, whereas HDACi or Rapa‐treated cells exhibited increased autolysosomes (mRFP^+^–mRFP^−^–LC3), suggesting that HDACi can facilitate autophagy‐inducing activity and autophagic flux (Figure 3H,I). Elevated levels of autophagy markers in cerebellar lysates of NPC1 KO mice were reduced in HDACi‐treated mice and SNAP25 was upregulated in the cerebellum of HDACi‐treated mice (Figure 3J,K). These results verified the effect of HDACi related to autophagic flux with SNAP25 upregulation in NPC1 KO mice, as well as in vitro using NPC‐iNSCs.

**FIGURE 3:**
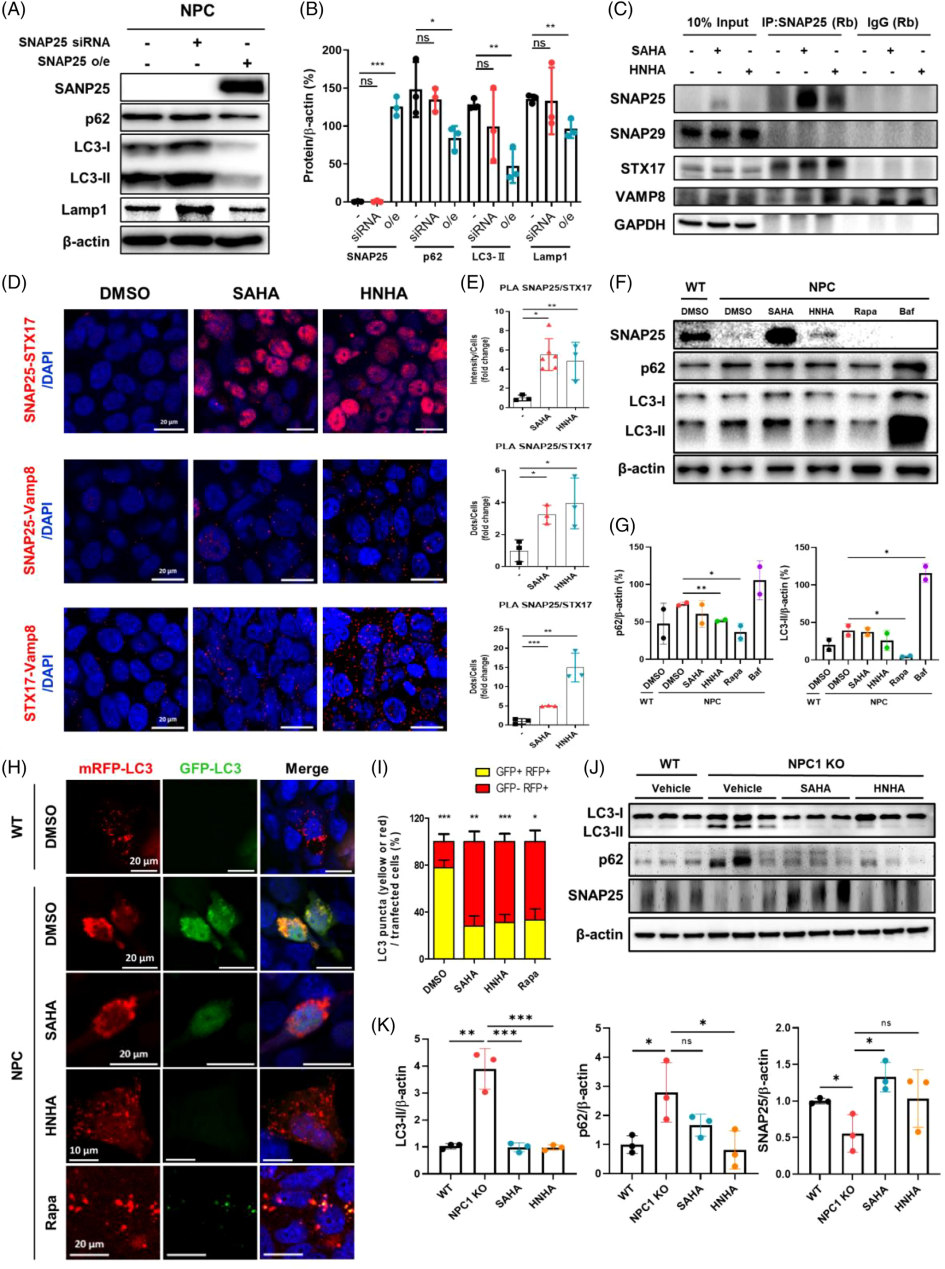
Upregulation of SNAP25 by suberoylanilide hydroxamic acid (SAHA) and *N*‐hydroxy‐7‐(2‐naphthylthio) heptanomide (HNHA) treatment is associated with autophagic flux in vitro and in vivo. (A) Niemann–Pick Type C disease (NPC)‐iNSCs were transfected with SNAP25 overexpression transcripts and siRNA targeting SNAP25 for 48 h, then lysed and subjected to western blotting. (B) Immunoblot band intensity was normalised to β‐actin. The graphs show the means ± SD (*n* = 3). (C) Immunoprecipitation of SNAP25 after 48 h of treatment with DMSO, SAHA (1 μM), or HNHA (1 μM), followed by co‐immunoprecipitation with STX17, SNAP29, and VAMP8 detected by western blotting. (D) NPC‐iNSCs treated with SAHA and HNHA for 48 h then processed using proximity ligation assay (PLA) assay. (E) The graphs show the means ± SD (*n* ≥ 3). (F) WT‐iNSCs, and NPC‐iNSCs were treated with SAHA (1 μM), HNHA (1 μM), Rapa (1 μM), or Baf (1 nM) for 48 h and then lysed and subjected to Western blotting. (G) Immunoblot band intensity was normalized to β‐actin expression. The graphs show the means ± SD (n = 2). (H) WT‐iNSCs and NPC‐iNSCs transfected with double‐tagged GFP‐mRFP‐LC3 and nontargeting scrambled RNA (scRNA) (1000 ng) for 24 h and then treated with SAHA (1 μM) and HNHA (1 μM), Rapa (1 μM), or Baf (1 nM) for 48 h. Representative images of cells under confocal microscopy. Scale bar, 10 or 20 μm as presented in each of image. (I) Graph showing the number of yellow (autophagosome) and red (autolysosome) puncta per cell. Graphs show the means ± SD (*n* = 10). (J) Representative Western blotting of the cerebellum in WT, NPC1 KO, SAHA and HNHA‐ treated mice with LC3, p62, and SNAP25 antibodies. (K) The quantification of Western blotting data of LC3‐II, p62, and SNAP25 in the cerebellum. Intensity was normalized to β‐actin expression. The graphs show the means ± SD (*n* = 3 biologically independent samples). Statistical significance was assessed by Student's *t*‐test. ***P < 0.001; **P < 0.01; *P < 0.05

Previous studies have reported that NPC‐iNSCs are defective in neuronal differentiation.^10^ NPC‐iNSCs exhibited reduced levels of TUJ1 (early neuron marker), neurofilament (NF), and MAP2 (mature neuron markers) compared to WT‐iNSCs. Notably, the number of TUJ1‐ and NF‐positive cells was significantly upregulated by HDACi‐treatment in NPC‐iNSCs (Figure 4A–F). In addition, SNAP25 overexpression could rescue the neuronal differentiation defects of NPC‐iNSCs by upregulating the level of NF in NPC‐iNSCs (Figure 4G,H). These results demonstrated that enriched SNAP25 via either pharmacological inhibition of HDAC or overexpression of SNAP25 alleviates defective neuronal differentiation in NPC‐iNSCs.

**FIGURE 4:**
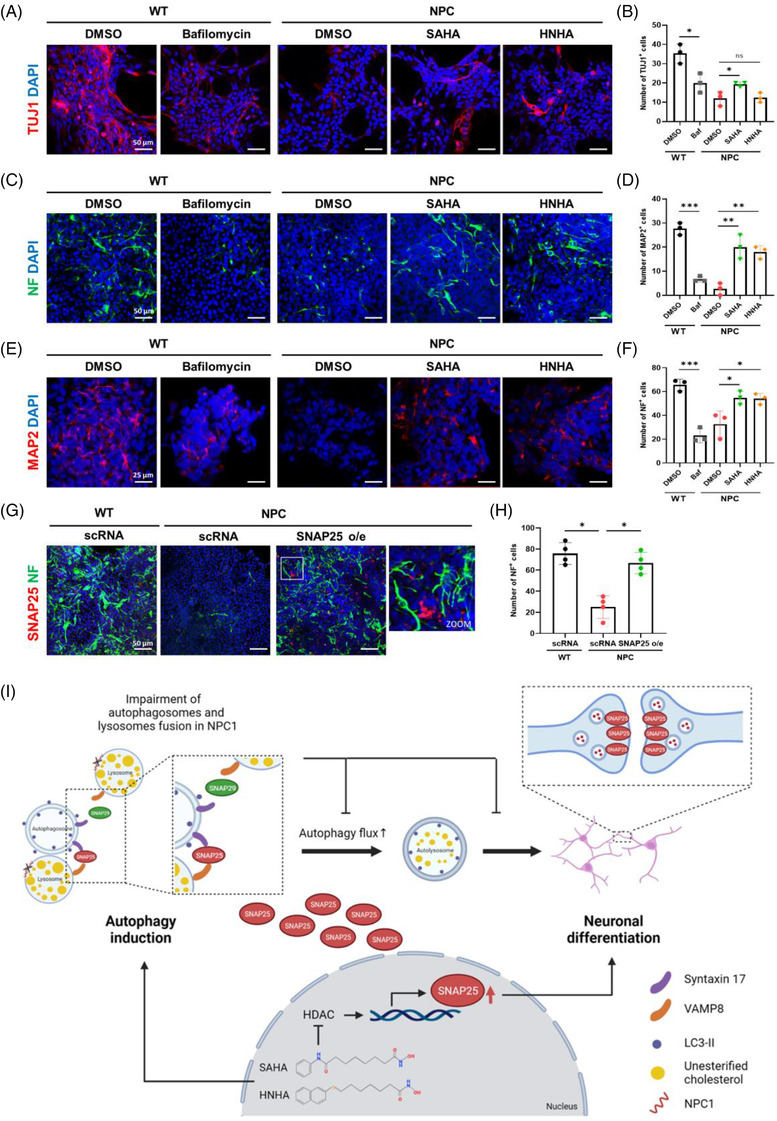
SNAP25 promotes the differentiation of Niemann–Pick Type C disease (NPC)‐iNSCs into neuronal cells. Representative images and quantification showing TUJ1 in WT‐iNSCs and NPC‐iNSCs after 7 days of neuronal differentiation. WT‐iNSCs and NPC‐iNSCs were treated with Bafilomycin, suberoylanilide hydroxamic acid (SAHA) and *N*‐hydroxy‐7‐(2‐naphthylthio) heptanomide (HNHA) for the indicated times. Scale bar, 50 μm. (B) Quantification of TUJ1 positive cells in WT‐iNSCs and NPC‐iNSCs. (C) Representative images and quantification showing NF in WT‐iNSCs and NPC‐iNSCs after 7 days of neuronal differentiation. WT‐iNSCs and NPC‐iNSCs were treated with Bafilomycin, SAHA and HNHA for the indicated times. Scale bar, 50 μm. (D) Quantification of NF positive cells in WT‐iNSCs and NPC‐iNSCs. (E) Representative images and quantification showing MAP2 in WT‐iNSCs and NPC‐iNSCs after 7 days of neuronal differentiation. WT‐iNSCs and NPC‐iNSCs were treated with Bafilomycin, SAHA and HNHA for the indicated times. Scale bar, 25 μm. (F) Quantification of MAP2 positive cells in WT‐iNSCs and NPC‐iNSCs. (G) Representative images of NF in WT‐iNSCs and NPC‐iNSCs after transfection with scRNA and SNAP25 overexpression. Scale bar, 50 μm. (H) Quantification of NF expression level in WT‐iNSCs and NPC‐iNSCs after transfection with SNAP25 overexpression. (I) Schematic summarizing the mechanism by which SAHA and HNHA attenuate NPC disease via inducing SNAP25‐mediated autophagy. The graphs show the means ± SD (*n* = 3). Statistical significance was assessed by Student's *t*‐test. ***P < 0.001; **P < 0.01; *P < 0.05

In conclusion, this study demonstrated that SNAP25 is a key player in alleviating the pathological phenotypes of NPC disease. Upregulation of SNAP25 by HDACi‐treatment rescued impaired autophagic flux and reduced abnormally accumulated cholesterol in NPC‐iNSCs cells via compensating for deficient STX17–SNAP29–Vamp8 complexes with STX17–SNAP25–Vamp8 complexes. Furthermore, increased SNAP25 recovered deficient neuronal differentiation capacity in NPC‐iNSCs and also improved survival of Purkinje cells in NPC1 KO mice, demonstrating that SNAP25 could enhance autophagy pathways and neuronal differentiation in vitro and in vivo (Figure 4I). Collectively, SNAP25 could be a novel therapeutic target for NPC disease since the upregulation of SNAP25 through HDACi treatment has shown both the increase of neuronal differentiation and the reduction of cholesterol accumulation.

## FUNDING

The National Research Foundation of Korea and government of the Republic of Korea, MSIP, Grant Numbers: 2015K1A1A2028365, 2018M3A9C4076477, 2021R1A3B1077371; the BK21 Yonsei Education & Research Center for Biosystems and Institute of Convergence Science (ICONS) at Yonsei University; Basic Science Research Program through the National Research Foundation of Korea (NRF) funded by the Ministry of Education, Grant Number: 2020R1A6A3A13069027; BK21 FOUR Future Veterinary Medicine Leading Education and Research Center at Seoul National University.

## Supporting information

Supporting information
