# Supplementary material for: Upregulation of SNAP25 by HDAC inhibition ameliorates Niemann‐Pick Type C disease phenotypes via autophagy induction
Source: Clin Transl Med. 2022 Apr 5;12(4):e776. doi: 10.1002/ctm2.776 (PMC8982502; doi:10.1002/ctm2.776)
Supplement: Supplementary file 1 — Supporting information [file CTM2-12-e776-s001.docx]

SUPPORTING INFORMATION FOR

**Upregulation of SNAP25 by HDAC inhibition ameliorates NPC disease phenotypes via autophagy induction**

Yooju Jung^1‡^, Seung-Eun Lee^2‡^, Insung Kang^2^, Sung Min Cho^1^, Kyung-Sun Kang^2^*, and Ho Jeong Kwon ^1^*

**MATERIALS AND METHODS**

**Induced neural stem cell culture**

Induced neural stem cells (iNSCs) were generated from normal human fibroblasts (GM05659; Coriell Institute for Medical Research) and NPC patient fibroblasts (GM03123^NPC1P237S/I1061T^, Coriell Institute for Medical Research) according to previous methods^1, 2^. In short, retroviral pMXSOX2 and pMX-HMGA2 were transfected into 293FT cells with VSV-G and gag/pol plasmids by the FuGENE 6 transfection reagent (Roche, Indianapolis, IN). At 72 h post-transfection, the supernatants were collected and used to infect human dermal fibroblasts with Polybrene (Sigma-Aldrich, Sigma, Ronkonkoma, NY). Following expansion of the infected cells, maintenance medium containing a 1:1 mixture of ReNcell medium (Millipore, Burlington, MA) and KnockOut DMEM/F-12 supplemented with StemPro NSC SFM Supplement (Gibco/Life Technologies, St. Petersburg, FL) with bFGF (Sigma-Aldrich, Saint-Louis, MO) and EGF (Sigma) was used for the iNSC culture. Cells were maintained as neurospheres and cultured in an attached state on a plate coated with Poly-L-ornithine (PLO) and fibronectin (FN) repeatedly. NPC-iNSC lines were generated using 10 independent clones. Represented results shown in main figures were from two different clones of GM03123 (#2 and #10). For neuronal differentiation, 5,000 iNSCs were seeded on PLO/FN-coated 24-well plate, and the medium was exchanged during a week.

**Cell proliferation assay**

Cells (3 × 10^3^ cells/well) were seeded onto 96-well plates (Tissue Culture Testplate, transparent and flat bottom, SPL Life Science, Korea) and incubated overnight prior to adding compounds for measuring proliferation. The cells were treated with compounds or siRNA for 24, 48, or 72 h, and cell proliferation was measured in triplicate using 3-(4,5-dimethylthiazol-2-yl)-2,5-diphenyltetrazolium bromide (MTT) at 0.4 mg/mL (final concentration).

**Neuronal differentiation *in vitro***

iNSCs at 5,000 cells per well were seeded onto PLO/FN-coated coverslips in 6-well plates and maintenance media containing a 1:1 mixture of ReNcell medium (Millipore, Burlington, MA) and KnockOut DMEM/F-12 supplemented with StemPro NSC SFM Supplement (Gibco/Life Technologies) with bFGF (Sigma-Aldrich) and EGF (Sigma) was used for 1 day. The next day, the medium was replaced with a neuron differentiation medium containing a 1:1 mixture of ReNcell media and KnockOut DMEM/F-12 supplemented with StemPro NSC SFM Supplement without any growth factors for random differentiation. Then, cells were treated with DMSO, SAHA 0.5 μM, HNHA 0.5 μM, and bafilomycin (baf) 1 nM for 8 days or transfected with siSNAP25 or SNAP25 overexpression vector for 2 days.

**Immunocytochemistry**

Cells were cultured in 24-well plates and washed 3 times with phosphate-buffered saline (PBS; Gibco) and fixed with 4% paraformaldehyde (PFA) in PBS for 10 minutes at room temperature. Fixed cells were permeabilized with 0.5% Triton X-100 for 10 minutes and then incubated with blocking solution containing 5% normal goat serum (NGS; Zymed, San Francisco, CA) for 1 h at room temperature. The dilution of primary antibodies **(Table S1)** was performed using the manufacturer’s recommended protocol, and cells were incubated overnight at 4℃. For secondary antibodies, Alexa 488- or Alexa 594-labeled antibodies (Invitrogen, Carlsbad, CA) were incubated for 1 h at room temperature. For nuclei staining, 4’,6-diamidino-2-phenylindole (DAPI; Sigma) was used for 10 minutes at room temperature. Images were captured with a confocal microscope (Nikon, Eclipse TE200, Tokyo, Japan).

**Filipin staining**

Cells were fixed with 4% paraformaldehyde for 10 minutes at room temperature. Fixed cells were boiled with antigen retrieval citrate buffer (10 mM sodium citrate and 0.05% Tween 20, pH 6.0) at 85°C for 10 minutes. Subsequently, cells were incubated with 100 μg/mL filipin (Cayman, Ann Arbor, MI) in PBS for 1 h. Images were captured using a confocal microscope (Nikon). The density of filipin staining in the cytoplasm was quantified using ImageJ software. More than three fields were analyzed for each condition, and graphs represent the average of the quantification.

**Cholesterol assay**

A cholesterol assay kit (Bio Vision, Milpitas, CA) was used to measure the free cholesterol level in cells. For sample preparation, 1x10^6^ cells were counted and suspended with 200 μL of cholesterol lysis buffer (chloroform: isopropanol: NP-40 (7:11:0.1)). Each sample was incubated overnight in a 50°C oven to remove chloroform. Dried samples were then dissolved in 200 μL cholesterol assay buffer by sonication until homogeneous. Cholesterol standardization was performed using the manufacture’s protocol. Subsequently, samples were mixed with reaction reagents from the cholesterol assay kit and incubated for 1 h at 37°C, protected from light. The absorbance of the assays was measured in a microplate reader using a wavelength of 570 nm.

**Next-generation sequencing analysis**

Four individual groups were collected and analyzed. Total RNA from each was isolated using TRIzol (Invitrogen) following the manufacturer’s protocol. Quantification and assessment of the condition of RNA was performed using a BioTek EpochTM spectrometer and Bioanalyzer 2100 system. ExDEGA (eBiogen, Seoul, Korea), an Excel-based differentially expressed gene analysis tool, was used to evaluate the differentially expressed genes, while clustering heat maps were generated using meV software. The p-values and false discovery rate (FDR) were calculated and assigned to each gene, and genes with fold change ≥2 and FDR < 0.05 were selected for further analysis. The National Center for Biotechnology Information Gene Expression Omnibus (GEO) accession number for the RNA-seq data reported in this paper is GSE185644.

**Proximity ligation assay (PLA)**

Proximity ligation assays between SNAP25-STX17, STX17-Vamp8, and Vamp8-SNAP25 proteins in HDACi-treated iNSC-NPCs were conducted according to the manufacturer’s instructions with mouse or rabbit anti-SNAP25, rabbit anti-STX17, and anti-Vamp8 antibodies (ab66066, ab5666, ab229646, and ab89158, Abcam). When each protein close to within 40 nm, single-stranded oligonucleotide, which are conjugated to each antibody, hybridize with the probes and amplify fluorescent-labelled oligonucleotides. Therefore, PLA assay provides visual detection and quantification of specific protein-protein interactions of interest in the cells. Duolink In Situ PLA Probe Anti-Mouse MINUS plus Anti-Rabbit PLUS (DUO92004 and DUO92002, Sigma-Aldrich), Duolink In Situ Detection Reagents Red (DUO92008, Sigma-Aldrich), and Duolink In Situ Wash Buffers, Fluorescence (DUO82049, Sigma-Aldrich). Cells were mounted with Duolink In Situ Mounting Medium with DAPI (DUO82040, Sigma-Aldrich) and observed under a Zeiss LSM 880 confocal microscope.

**Co-immunoprecipitation**

Sub confluent cells were cultured with 1 μM SAHA, 1 μM HNHA, or 0.1% DMSO for 48 h. Cells were washed with PBS and lysed with lysis buffer (50 mM Tris-HCl pH 7.4, 150 mM NaCl, 1% NP-40, and 1 mM EDTA) containing Halt Phosphatase and Phosphatase Inhibitor Cocktail (78446, Thermo Scientific). Anti-SNAP25 antibody 2.5 μg (ab5665, Abcam) or anti-IgG rabbit isotype control 2.5 μg (3900S, Cell Signaling) were incubated with pre-cleared Dynabeads Protein A (10006D, Invitrogen) for 1 h at 4℃. Antibody-conjugated magnetic beads were washed three times with lysis buffer. Cell lysates were incubated with antibody-conjugated magnetics beads overnight at 4℃ with rotation and then washed three times with lysis buffer. Pellets were resuspended in 1× SDS sample buffer and boiled for 10 minutes at 100℃. Samples were then applied to SDS-polyacrylamide gel electrophoresis (SDS-PAGE) for western blotting.

**SDS-PAGE and immunoblotting**

Cells were homogenized in SDS with Complete Protease Inhibitor Cocktail and PhosSTOP Phosphatase Inhibitor Cocktail (Roche Life Sciences, Indianapolis, IN) and separated by 8%–15% SDS-PAGE. Proteins were transferred with 2.275 g/L Tris and 7.5 g/L glycine to PVDF membranes and incubated overnight at 4°C with primary antibodies **(Table S1)**. The membrane was then incubated with rabbit or mouse secondary antibody (1:3000 v/v) in 3% skim milk or 3% BSA for 1 h at room temperature (RT). Immunolabeling was detected using an enhanced chemiluminescence kit (GE Healthcare, Chicago, IL) according to the manufacturer’s instructions and a ChemiDoc XRS+ imaging system (BioRad, Hercules, CA).

**Immunofluorescence**

Cells cultured on coverslips were fixed with 4% paraformaldehyde for 10 minutes and permeabilized with 0.1% Triton X-100. After blocking in blocking buffer (3% BSA; Sigma, #A2153) in PBS, cells were treated with primary antibodies **(Table S1)**. Following incubation at 4°C overnight, cells were washed with PBS and incubated with secondary antibodies conjugated to Alexa Fluor 594 or Alexa Fluor 488 for 1 h at RT. Nuclei were stained with Hoechst 33342 (Thermo Fisher Scientific). Cells were washed with PBS, mounted, and observed under a Zeiss LSM 880 confocal microscope.

**mRFP-GFP-LC3 plasmid, siRNA, and SNAP25 overexpression vector transfection**

iNSCs were seeded in 6-well plates at 2.0 × 10^5^ cells/well and incubated overnight. iNSCs were then transfected overnight with control or mRFP-GFP-LC3 plasmids using Lipofectamine LTX transfection reagent (Invitrogen, 94,756). The cells were treated with SAHA, HNHA, Rapamycin (Rapa), and Bafilomycin (Baf) for 48 h, followed by treatment with mRFP-GFP-LC3 plasmid (1000 ng). The cells were fixed with 4% formaldehyde (Sigma-Aldrich, 252,549; diluted in PBS) and washed three times with 1× PBS (CureBio, P0213; diluted in distilled water). Nuclei were stained with Hoechst (Sigma-Aldrich, 33342) following incubation for 10 minutes and washed three times with 1× PBS. Images were obtained using a confocal microscope at a 400× magnification. siRNAs targeting human SNAP25 transcripts and scrambled/non-targeting siRNA were purchased from Dharmacon (L-011394-01-0005 and D-001810-10-05, sequence data are presented in **Table S2**) and reconstituted with nuclease free water. iNSCs were transfected with 50 nM SNAP25 siRNA overnight then treated with compounds for 48 h. Human SNAP25 ORF mammalian expression plasmid and SNAP25 (GFP-tagged) human SNAP25 transcripts were purchased from Sino Biological, Inc. (HG11585-NF, Wayne, PA) and OriGene Technologies (RG202068, Rockville, MD). Plasmids were reconstituted and amplified according to the manufacturers’ instructions. iNSCs were transfected overnight with 1000 ng of plasmids. We used a tandem fluorescent-tagged mRFP-GFP-LC3 reporter to assess autophagic flux. Because GFP signal is quenched in acidic lysosomes, autolysosomes emit an acid-stable mRFP (red) signal showing that autophagic flux is functional. Conversely, when autophagic flux is blocked, autophagosomes emit both mRFP/GFP (yellow) signals.

**Nissl staining**

For cresyl-violet (Nissl) staining, whole-brain slices were mounted on slides and dried overnight. Mounted sections were soaked with 100%, 90%, and 70% ethanol for 5 min at room temperature, then rinsed with distilled water. Nissl staining was performed by soaking the samples in pre-warmed 0.1% Nissl violet solution for 10 min. After a brief rinse with distilled water, samples were transferred to 70%, 90%, and 100% ethanol each for 1 minute, then dehydrated in 100% ethanol. Samples were mounted using Canada balsam after a xylene-clearing step.

**Quantitative reverse transcriptase-PCR (qRT-PCR)**

Total RNA was extracted from cells or tissues using TRIzol (Invitrogen) according to the manufacturer’s instructions. Five hundred nanograms of RNA were reverse-transcribed into complementary DNA (cDNA) using the Superscript First-Stand Synthesis System (Invitrogen). Quantitative real-time PCR was performed using SYBR Green PCR master mix (Applied Biosystems, Foster City, CA), and the expression of mRNA level was normalized to that of the housekeeping gene GAPDH. Primer sequences were as follows: *GAPDH* (F: 5’- TGC CCA GAA CAT CAT CCC TG -3’ R: 5’- GAC GGA CAC ATT GGG GGT AG -3’), *SNAP25* (F: 5’– ACC AGT TGG CTG ATG AGT CG- 3’ R: GTT CGT CCA CTA CAC GAG CA- 3’), SNAP29 (F: 5’- GGC AGC AGT ACC TGA GAC AG-3’ R: 5’-AGA GGC GAC TCC GAT CTT CT-3’), *LAMP1* (F: 5’- CTT CAG GCC ACT GTG GGA AA -3’ R: 5’- CCT GGA CCT GCA CAC TGA AG -3’), *LAMP5* (F: 5’- CCC AAT CCC ATT GGC CTT CT -3’ R: 5’ - GGA AGC ACC CAG GAT GCA G -3’).

**Animals**

A breeding pair of heterozygous Balb/c NPC1+/- mice was purchased from Jackson Laboratories (Farmington, CT). Wild-type (NPC1+/+) and NPC1-knockout mice (NPC1-/-) were used for *in vivo* studies and heterozygous mice were kept for further breeding. Genotyping was performed with genomic DNA isolated from tail snips using PCR-based protocols suggested by Jackson Laboratories. All animal experiments were performed in accordance with regulations established by Seoul National University Institutional Animal Care and Use Committee.

**Rota rod test**

Balance and motor coordination of mice were evaluated using rotarod treadmill tests (7650 Accelerating model, Ugo Basile Biological Research Apparatus, Comerio, Italy). Rota-rod tests were performed two times a week on mice treated with compounds for 2 weeks approximately 5 to 8 weeks prior. The electronically controlled rotating rod accelerates at a constant speed of 30 rpm over 3 minutes and runs for three trials per test. At 4 weeks of age, the mice were trained for 1 week prior to having their motor function tested at a speed of 10 rpm. The representative record of each subject was acquired as the mean performance time of all three attempts.

**Tissue preparation and histological analysis**

For histological analysis, mice were perfused with saline and 4% paraformaldehyde (PFA) at 8 weeks of age. Whole-brain tissue was fixed in 4% PFA at 4℃ for a day and then stored in 30% sucrose until they sank. For immunohistochemical analysis, brain tissues were cryopreserved with OCT compound (Sakura Finetek, Tokyo, Japan) and stored overnight at -80℃. Cryo-embedded samples were sectioned into 20-µm serial transverse sections, then washed several times with PBS and permeabilized with 0.02% Triton X-100. After permeabilizing, the sections were incubated in 5% normal goat serum for blocking process and incubated with primary antibodies **(Table S1)** overnight at 4℃. Samples were then incubated with Alexa 488- or 594-labeled secondary antibodies (Invitrogen) for 1 h at room temperature after PBS washing. Finally, DAPI (Zymed Laboratories, Inc.) staining was used to visualize the cell nuclei.

**Statistical analysis**

The mean values of all results are presented as the mean ± SD. All samples were randomly selected and analyzed. Statistical analyses were performed using two-tailed Student’s *t*-test or ANOVA followed by Bonferroni’s test for multiple comparisons. *P*-value of less than 0.05 was considered statistically significant (*indicates *P*< 0.05; **indicates *P*< 0.01; ***indicates *P*< 0.001). Statistical analyses were performed using Prism version 7.0 (GraphPad Software, San Diego, CA).

**DISCUSSION**

Our findings revealed that upregulation of SNAP25 by HDAC inhibition ameliorates NPC disease phenotypes via enhancing autophagy flux and neuronal differentiation in NPC-iNSCs. To investigate the effects of SAHA and HNHA treatment in NPC disease, we treated iNSCs from NPC1 patient-derived fibroblasts as described in our previous study. Terminally differentiated neurons derived from iNSCs are very useful for understanding the pathophysiological mechanisms of disease, so by doing this, we were able to mirror the pathological mechanisms of NPC disease *in vitro*.

Based on structural differences between SAHA and HNHA, it is unsurprising that their bioactivity mechanisms to reduce free cholesterol were shown to be slightly different. As shown on **Fig. S1A**, HNHA has a diphenyl structure instead of phenyl in cap part of the compound, causing it to have increased hydrophobic properties compared to SAHA. The linker part of HNHA consists of a sulfide group instead of an amino group, which may help HNHA more easily penetrate through membranes, thus increasing its drug efficacy. Lastly, SAHA and HNHA were evaluated using Lipinski’s rule of five. The molecular weight of both compounds are less than 500 kDa, and the logP values of SAHA and HNHA are 1.9 and 4.1, respectively. Because a logP value of approximately 4 is usually considered to show the most ideal ‘druglikeness’, HNHA is more likely to be a druglikeness small molecule than SAHA, meaning that HNHA will likely have better efficacy than SAHA. However, in the case of this study, HNHA showed lower bioactivity than SAHA in NPC-iNSCs, likely because of differences in multi-target or off-target effects between the two compounds. In future studies, we aim to determine whether the detailed mechanism of action of the two compounds is different.

Previously, SAHA and HNHA have been reported that they acetylated histone 3 (H3) and tubulin respectively^3,4^, we investigated whether two compounds also acetylate H3 and tubulin in NPC-iNSCs or not before conducting NGS analysis. As the result, SAHA and HNHA upregulated acetyl-H3 and acetyl tubulin in NPC-iNSCs, indicating that SAHA and HNHA inhibited HDAC activity in the cells and could regulate HDAC associating with acetyl-H3 (Fig. S2A-C). In Fig. 2E and F, because the differences in the SAHA-treated gene profiles showed a larger difference in gene expression than the HNHA-treated gene profiles, the top three categories that increased the most based on the SAHA group were selected, including P; Inorganic ion transport and metabolism (SAHA: 8.77, HNHA: 4.91), Z; Cytoskeleton (SAHA: 7.89, HNHA: 5.06), and U; Intracellular trafficking, secretion and vesicular transport (SAHA: 7.02, HNHA: 4.83). We decided to perform a more in-depth analysis of the factors for category U. Therefore, to identify the mutual targets of SAHA and HNHA, we generated a heat map based on category U associated genes on DMSO, SAHA, or HNHA treated NPC-iNSCs and selected SNAP25 as the same target of two compounds.

Interestingly, when SNAP25 was endogenously knocked down with siSNAP25, the average filipin intensity of WT-iNSCs was unchanged compared to scRNA-transfected WT-iNSCs as shown in **Fig. 2F**. This is probably because the SNARE machinery, meaning STX17-SNAP29-VAMP8, is functioning normally in WT-iNSCs, so downregulation of SNAP25 has little effect. SNAP29 is also a SNARE protein that regulates autophagic flux and membrane trafficking^5^.

In Fig. 3E-F, Rapa significantly decreased p62 and LC3-Ⅱ more accumulated by Baf, but SNAP25 did not increase. Based on these results, SAHA and HNHA show autophagy activity through overexpression of SNAP25 unlike Rapa, which is a conventional autophagy inducer via mTOR regulation.

We also examined whether SNAP25 has a therapeutic effect on NPC1 KO mice. By demonstrating the positive effect of SNAP25 on cholesterol regulation in NPC-iNSCs and that the expression of SNAP25 protein was decreased in NPC1 KO mice, we speculate that the neuropathological phenotypes of NPC disease may be caused by altered expression of SNAP25 in the brain. It is known that SNAP25 promotes exocytosis of neurotransmitters during synaptic neurotransmission^6^. The baseline expression level of SNAP25 in the cerebellum was significantly higher than that of the liver because SNAP25 is highly expressed in neuronal cells. Despite showing the effects of SAHA and HNHA at the cellular level, treatment of both SAHA and HNHA did not extend the lifespan in the NPC1 KO mouse model because of their poor ability to penetrate the BBB. Alam MS *et al.* previously reported that SAHA alone showed bioactivity in primary cells derived from mice but did not prolong animal survival because SAHA could not cross the BBB^7^. To address this problem, a triple combination formulation containing SAHA was given to NPC1 KO mice, resulting in extending mouse life span. In future studies, we intend to apply this novel delivery system when administering HNHA *in vivo*.

To characterize which class of HDAC regulates increased the level of SNAP25 by SAHA and HNHA treatment, HDAC class Ⅰ specific inhibitor FK228 and HDAC class Ⅱ specific inhibitor tubacin were treated on NPC-iNSCs. FK228 increased SNAP25 significantly reducing p62 and tubacin increased SNAP25 similar to HNHA (Fig. S3E-F). Based on the following results, it is thought that the expression of SNAP25 is more regulated by HDAC class Ⅰ rather than HDAC class Ⅱ, however additional studies are imperative.

Autophagy is a critical process in NPC disease, and dysregulated autophagic flux in NPC disease is associated with impaired autophagosome-lysosome fusion leading to Purkinje cell loss^8^. Our study of the SNAP family strongly suggests that SNAP25-mediated protective effects include modulation of the autophagic process, which in turn promotes degradation activity within the autolysosome **(Fig. S4A)**. The current data show that SNAP25 is increased by SAHA and HNHA treatment in NPC models, which could reduce the pathological changes associated with NPC disease by upregulating the normal autophagic process. In addition, the levels of lysosomal markers, LAMP1 and LAMP5, were also decreased by SAHA and HNHA treatment as shown in **Fig. S4B**, suggesting that upregulated SNAP25 influences the autophagic degradation process in NPC disease. Taken together, we conclude that SNAP25 may be dysregulated in NPC disease linked to defects in autophagic pathways and cholesterol homeostasis and could be a critical target or biomarker for drug therapy in NPC disease.

**SUPPLEMENTAL FIGURES**

**
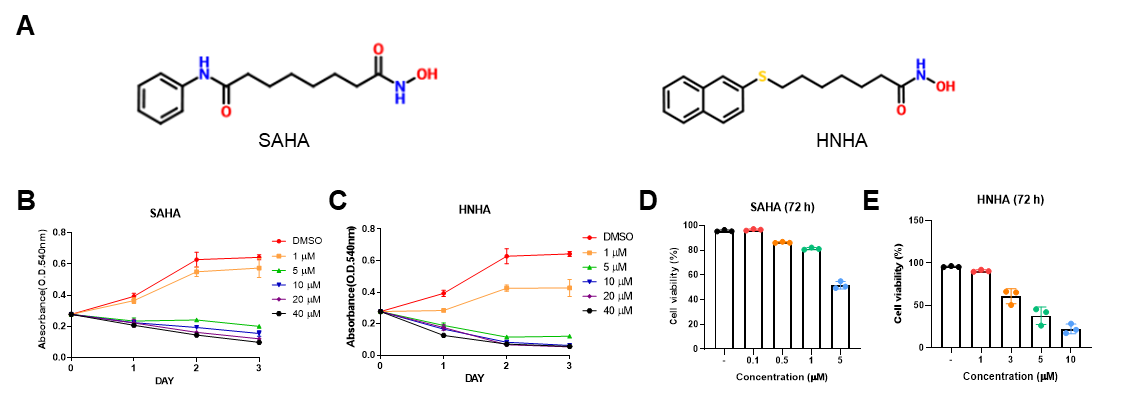
**

**Figure S1. Chemical structure of HDAC inhibitors and their effect on the proliferation of NPC-iNSCs.** (A) Chemical structures of SAHA and HNHA. (B, C) Effect of the HDAC inhibitors SAHA and HNHA on the proliferation of NPC-iNSCs. NPC-iNSCs were treated with various concentrations of SAHA and HNHA (0–40 µM) for 72 h in triplicate, and their proliferation was measured by MTT assay. (D, E) The viability of SAHA- and HNHA-treated NPC-iNSCs at various concentration was measured using a trypan blue assay.


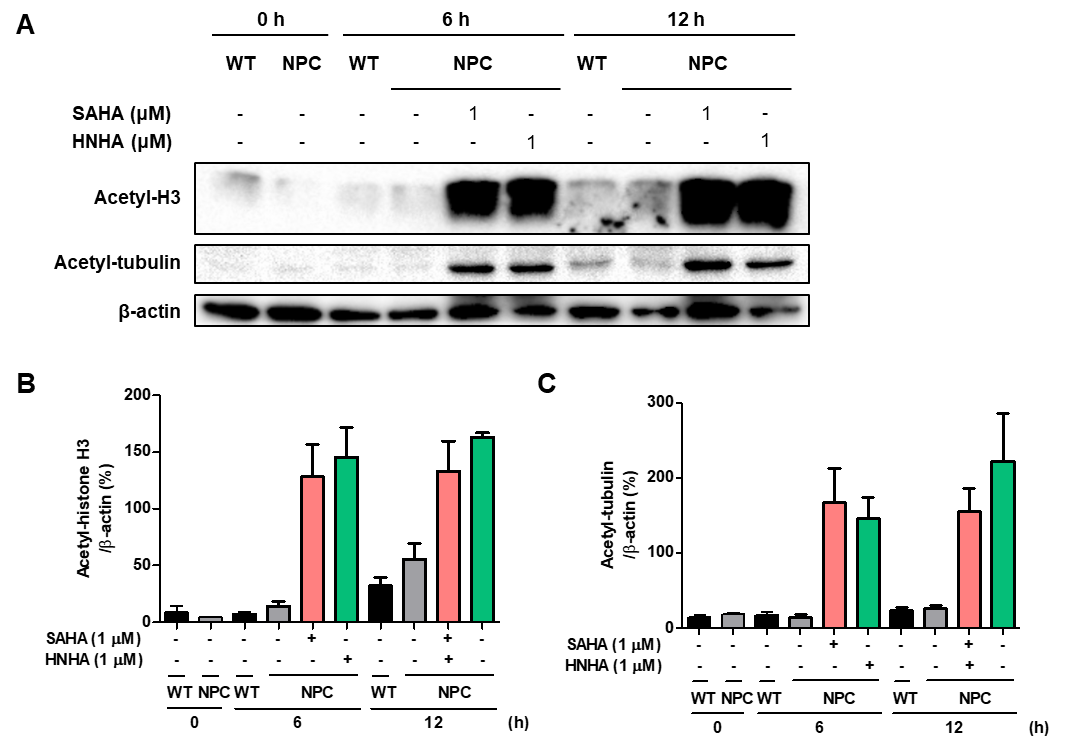


**Figure S2. SAHA and HNHA acetylated Histone H3 and tubulin.** (A) WT-iNSC treated with DMSO and NPC-iNSCs treated with DMSO, SAHA (1 μM), and HNHA (1 μM) for 0, 6, and 12 h. Cell extracts were subjected to immunoblotting with acetyl-H3, acetyl-tubulin and β-actin. (B), (C) Immunoblot band intensity normalized to β-actin. The graphs show the means ± SD (n = 2).


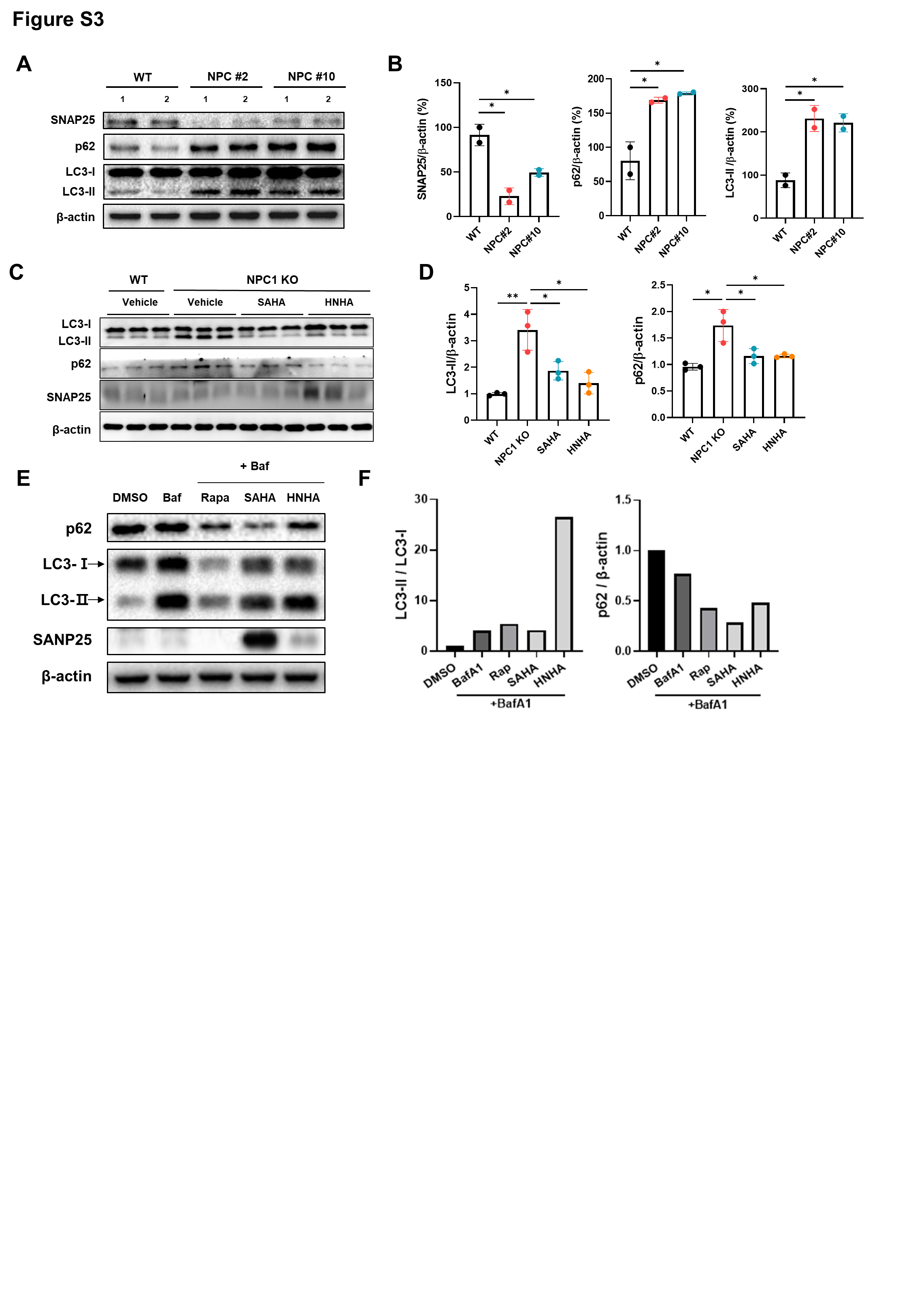


**Figure S3. SNAP25 mediates autophagic flux *in vitro* and *in vivo*.** (A) WT-iNSCs, NPC-iNSCs #2, and NPC-iNSCs #10 were lysed and subjected to western blotting with LC2 and p62 antibodies. (B) Protein expression of SNAP25, p62, and LC3-II normalized to that of β-actin. The graphs show the means ± SD (n = 2). (C) Western blotting of liver samples in WT and NPC1 KO mice with LC3, p62, and SNAP25 antibodies. (D) The quantification of western blotting data of LC3-II, p62 and SNAP25 in liver samples. The intensity of the bands was normalized to β-actin expression. (E) WT-iNSCs, and NPC-iNSCs treated with SAHA (1 μM), HNHA (1 μM), Rapa (1 μM), and Baf (1 nM) with or without Baf (1 nM) for 48 h and then were lysed and subjected to western blotting. (F) The quantification of western blotting data of LC3-II/LC3-I, and p62/β-actin. Statistical significance was assessed by Student’s *t*-test. ****P*< 0.001; ***P*< 0.01; **P*< 0.05.


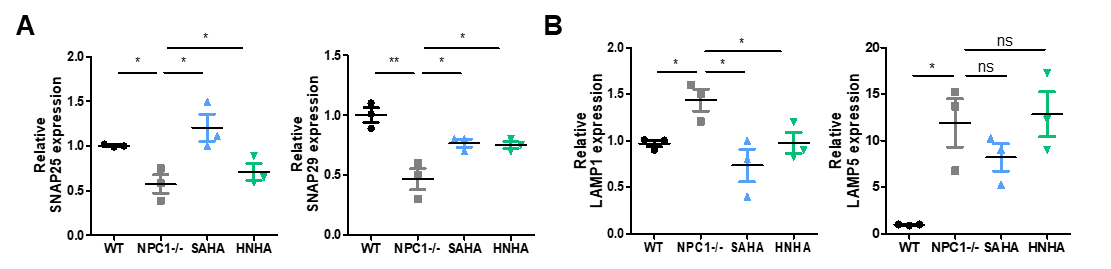


**Figure S4. The expression level of lysosome and vesicle-associated membrane marker in each cerebellar sample.** (A) mRNA levels of SNAP25 and SNAP29 in cerebellar samples using qRT-PCR. (B) mRNA levels of lysosome associated marker (LAMP1, LAMP5) were examined in each cerebellar samples using qRT-PCR. GAPDH was used for loading control and the expression levels of genes were normalized to WT-iNSCs. Statistical significance was assessed by Student’s t-test. **P < 0.01; *P < 0.05.


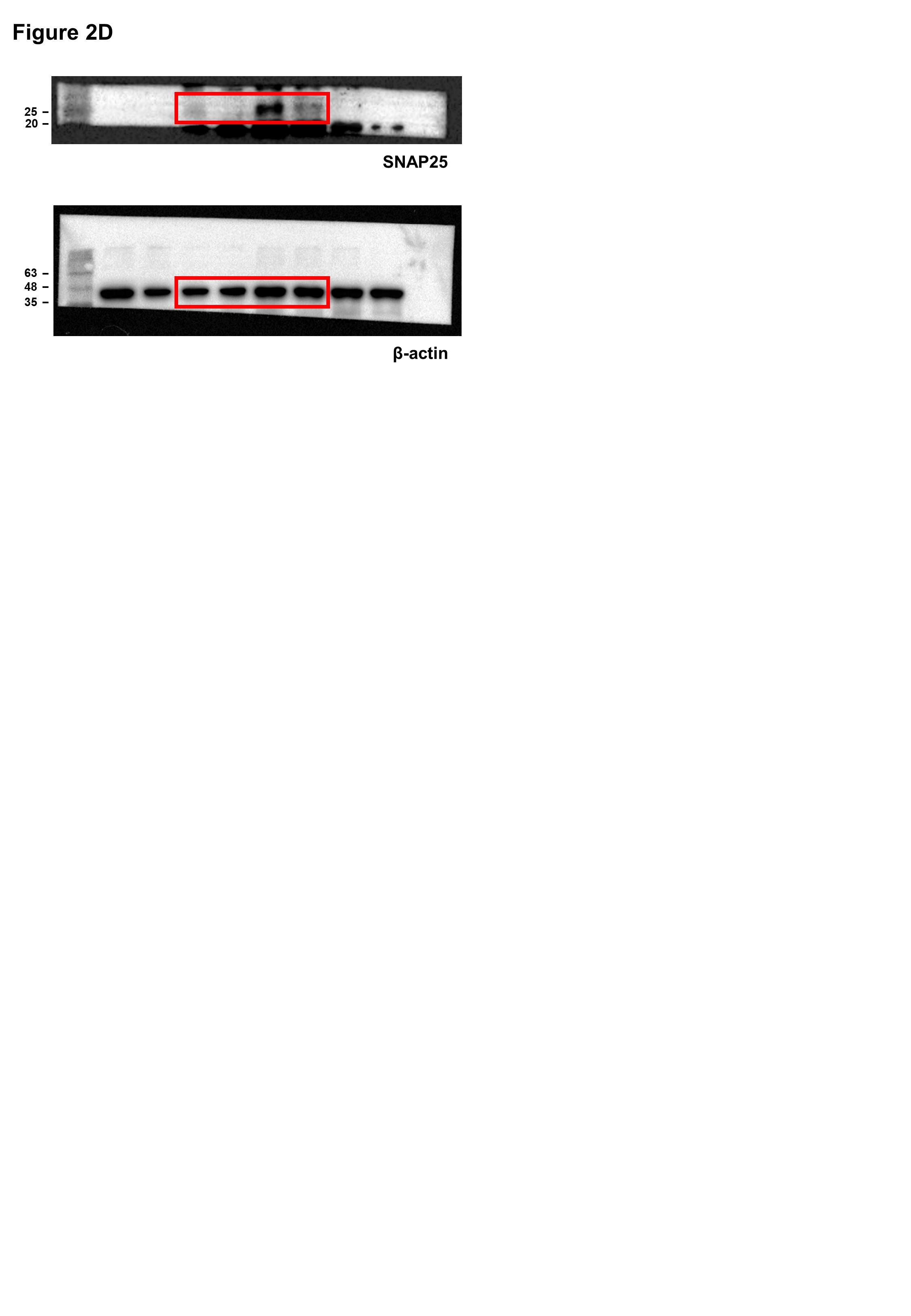


**Figure S5. Original unedited data shown in Figure 2. Each figure corresponds to the immunoblots with the figure number shown.**

**Figure S6. Original unedited data shown in Figure 3. Each figure corresponds to the immunoblots with the figure number shown.**


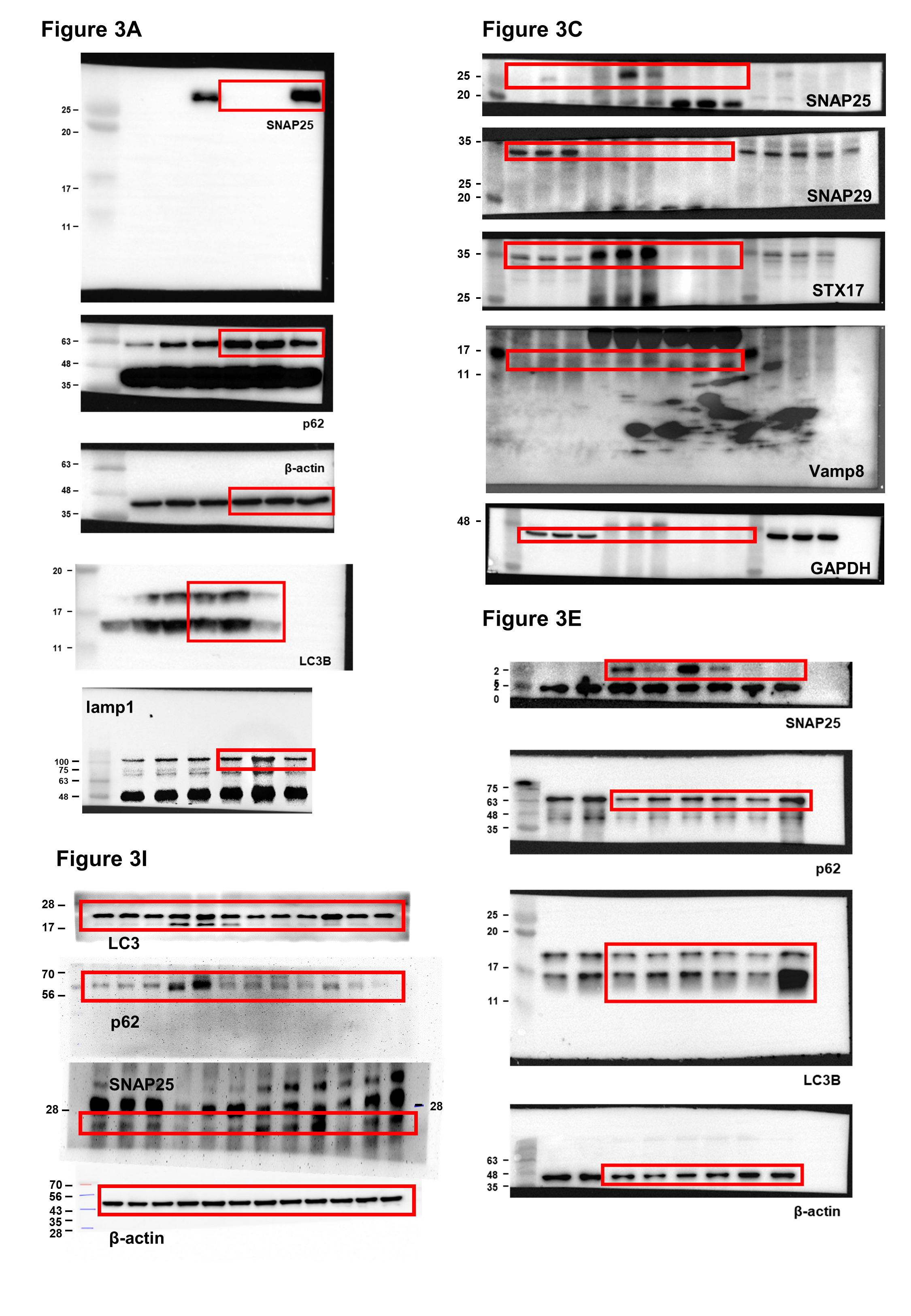


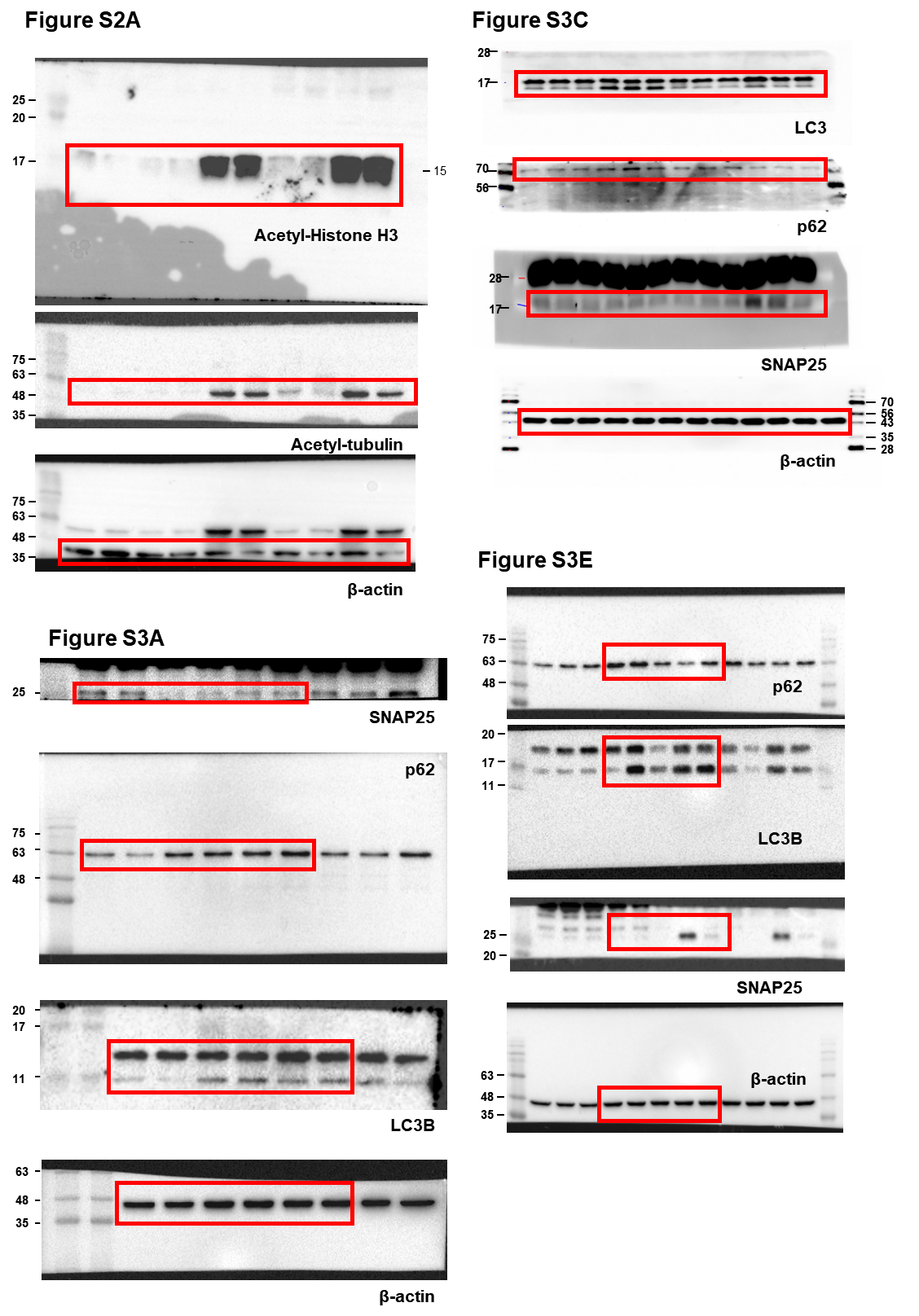


**Figure S7. Original unedited data shown in Supplementary figure S2 and S3. Each figure corresponds to the immunoblots with the figure number shown.**

**Table S1 List of antibodies used in this study**

| **Name** | **Species** | **Dilution** | **Vendor** | **Catalog #** |
| --- | --- | --- | --- | --- |
| SNAP25 | mouse | 1:1000 | abcam | ab 66066 |
| SNAP25 | Rabbit | 1:1000 | abcam | ab 5666 |
| SNAP29 | Rabbit | 1:1000 | GeneTex | ab220873 |
| STX17 | Rabbit | 1:1000 | abcam | ab229646 |
| VAMP8 | Rabbit | 1:1000 | Biolegend | ab76021 |
| VAMP8 | mouse | 1:1000 | abcam | ab 89158 |
| LAMP1 | mouse | 1:1000 | sigma | ab25630 |
| LC3B | Rabbit | 1:1000 | abcam | ab48394 |
| p62 | mouse | 1:1000 | Cell signaling | BD610833 |
| Acetyl-Histone H3 | Rabbit | 1:1000 | Thermo Fisher | PA5-32028 |
| Neurofilament L | Rabbit | 1:500 | abcam | ab 9568 |
| Tubulin β 3 (TUBB3) | Mouse | 1:500 | Cell signaling | B249869 |
| NPC1 | Rabbit | 1:1000 | abcam | ab108921 |

**Table S2 Human SNAP25 siRNA sequence data**

| **L-011394-01-0005 ON-TARGET plus Human SNAP25 (6615) siRNA** |
| --- |
| ON-TARGETplus SMARTpool siRNA J-011394-09, SNAP25  Target Sequence: CUGGAAAGCACCCGUCGUA |
| ON-TARGETplus SMARTpool siRNA J-011394-10, SNAP25  Target Sequence: CAGAAUCGCCAGAUCGACA |
| ON-TARGETplus SMARTpool siRNA J-011394-11, SNAP25  Target Sequence: GUGUAGUGGACGAACGGGA |
| ON-TARGETplus SMARTpool siRNA J-011394-12, SNAP25  Target Sequence: ACAAAUGAUGCCCGAGAAA |

**REFERENCES**

1. Yu, K.R.*, et al*. Rapid and Efficient Direct Conversion of Human Adult Somatic Cells into Neural Stem Cells by HMGA2/let-7b. *Cell rep*. **10**, 441-452 (2015).

2. Sung, E.A.*, et al*. Generation of patient specific human neural stem cells from Niemann-Pick disease type C patient-derived fibroblasts. *Oncotarget*. **8**, 85428-85441 (2017).

3. Huang, HL.*, et al*. Anticancer activity of MPT0E028, a novel potent histone deacetylase inhibitor, in human colorectal cancer HCT116 cells in vitro and in vivo. *PLoS one*. **7**, e43645 (2012).

4. Kim, SM.*, et al*. Potential anti-cancer effect of N-hydroxy-7-(2-naphthylthio) heptanomide (HNHA), a novel histone deacetylase inhibitor, for the treatment of thyroid cancer. *BMC cancer*. **15**, 1003 (2015).

5. Morelli, Elena, et al. Multiple functions of the SNARE protein Snap29 in autophagy, endocytic, and exocytic trafficking during epithelial formation in Drosophila. *Autophagy*. 10(12), 2251-2268 (2014).

6. Antonucci, F.*, et al*. SNAP-25, a Known Presynaptic Protein with Emerging Postsynaptic Functions. *Front synaptic neurosci*. **8**, 7 (2016).

7. Alam, M.S., Getz, M. & Haldar, K. Chronic administration of an HDAC inhibitor treats both neurological and systemic Niemann-Pick type C disease in a mouse model. *Sci transl ned*. **8**, (2016).

8. Sarkar, S.*, et al*. Impaired autophagy in the lipid-storage disorder Niemann-Pick type C1 disease. *Cell rep*. **5**, 1302-1315 (2013).
